# Improved control over implant anchorage under the use of the femoral neck system for fixation of femoral neck fractures: a technical note

**DOI:** 10.1186/s12891-021-04497-x

**Published:** 2021-07-13

**Authors:** Yonghan Cha, Ji-Ung Song, Jun-Il Yoo, Ki Hoon Park, Jung-Taek Kim, Chan Ho Park, Won-Sik Choy

**Affiliations:** 1 Department of Orthopaedic Surgery, Eulji University Hospital, Daejeon, South Korea; 2 Department of Orthopaedic Surgery, Chamjoen Hospital, Gwangju, South Korea; 3 Department of Orthopaedic Surgery, Gyeongsang National University Hospital, Jinju, South Korea; 4 Department of Orthopedic Surgery, Ajou University School of Medicine, Ajou Medical Center, 164, World cup-ro, Yeongtong-gu, 16499 Suwon-si, Gyeonggi-do South Korea; 5 Department of Orthopaedic Surgery, Yeungnam University Medical Center, Daegu, South Korea

**Keywords:** Femoral neck fracture, Treatment, Fixation, Femoral neck system, Hip

## Abstract

**Background:**

The depth of bolt in Femoral neck system (FNS, DePuy Synthes, Oberdorf, Switzerland) is difficult to finely control as the length of the bolt is in units of 5 mm. Thus, this study introduces a method to control the depth of FNS bolt in analogue scale in patients with femoral neck fracture.

**Methods:**

By the technique of control of reaming and retraction of bolt, the tip of implant could be positioned close to subchondral bone without harming it. The position of implant tip in four cases in which the introduced technique was applied was compared to that of eight cases where the standard technique was performed.

**Results:**

The average tip-apex distance measured in the cases that underwent surgery using the suggested technique in this study was statistically significantly shorter than that measured in the cases that underwent surgery under manufacturer guidelines.

**Conclusion:**

Even though the bolt of FNS is manufactured in the unit of 5 mm, the technique proposed in this study helps surgeons to adjust the depth of bolt for the fixation of femoral neck fracture using FNS.

## Background

The surgical treatment of femoral neck fractures can be classified into internal fixation and arthroplasty. In order to determine the surgical method, various factors such as the patient's age, bone density, fracture dislocation, and type of fracture must be considered [1]. Among them, internal fixation is preferred for undisplaced fracture or for relatively young patients, and either closed or open reduction can be performed [2–4]. Implants for internal fixation in femoral neck fracture include cannulated screws, dynamic hip screw (DHS) with or without antirotation screw, DHS with blade instead of screw or similar implants [5–7]. Parallel multiple cannulated screws (MCS) are commonly used in relatively young patients with femoral neck fractures, but they are associated with lower construct stiffness and earlier failure under cyclic loadings compared with DHS, as demonstrated in previous biomechanical tests [8–10]. Although DHS provides more stability to femoral neck fractures than MCS, it requires larger skin incision and more extensive soft tissue dissection [11].

**Fig. 1:**
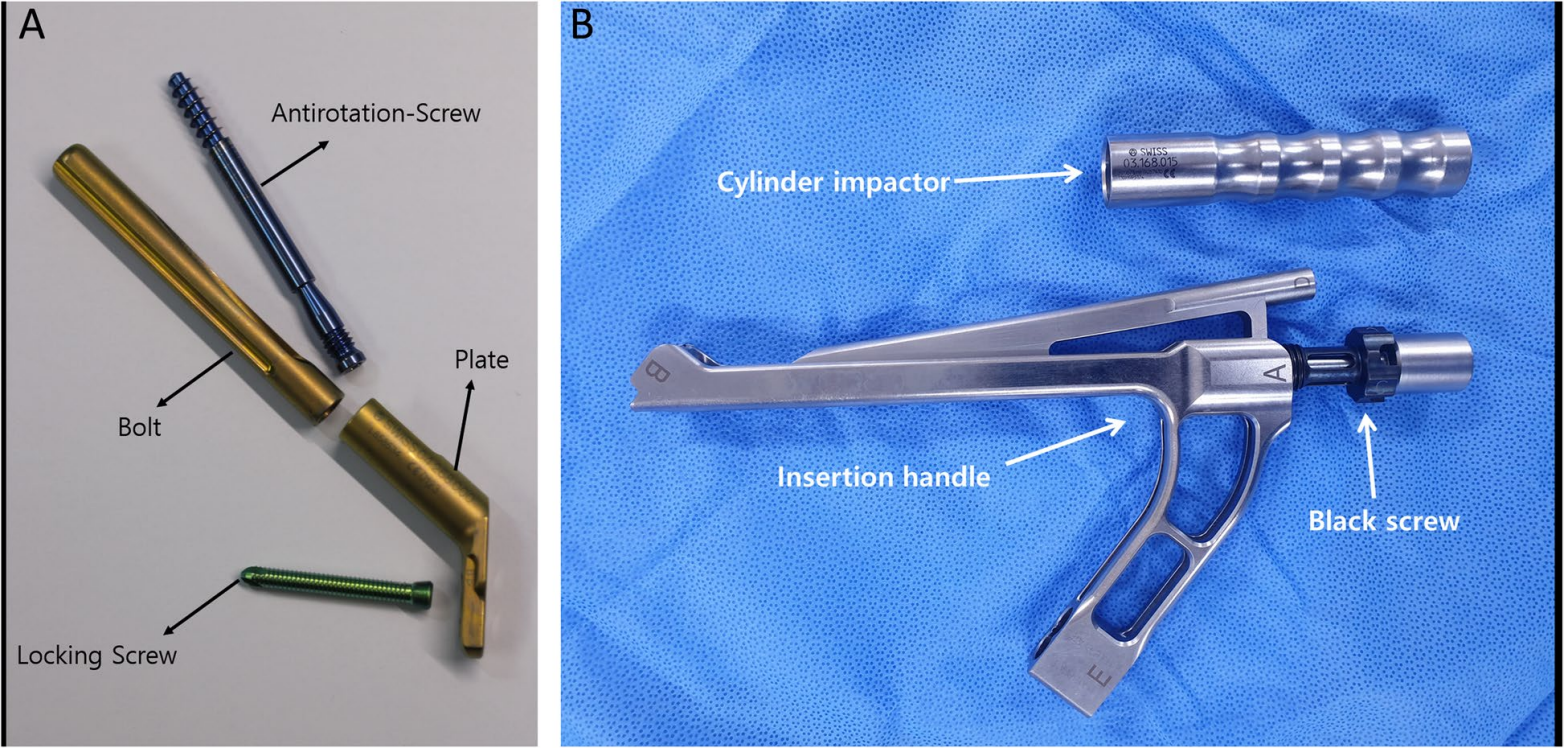
**A** Femoral neck system consists of bolt, plate, antirotation screw and locking screw. **B** Insertion handle and cylinder impactor. The photographs were taken by the authors

The recently introduced implant, the Femoral Neck System (FNS, DePuy Synthes, Oberdorf, Switzerland) (Fig. 1), has both advantages of above two implants. It requires small incision like MCS and provides angular stability like DHS [9].

The FNS is composed of (1) a plate with a barrel and threaded screw holes which accommodates 1 or 2 locking screws, (2) an antirotation screw, and (3) a bolt that supports the head fragment. The proximal fragment with femoral head is held tight by the bolt and antirotation screw, thus it can slide through the axis of barrel to obtain controlled dynamic compression of the fracture site (Fig. 1).

Comparing an extremely robust metal to a fractured bone, acquisition of a longer moment arm provides more stability after implant fixation [9]. As penetration of implant out of femoral head surface jeopardizes the uninjured joint, surgeons walk a tightrope between violating the joint and gaining stability.

The depth of implant insertion of DHS and multiple cannulated screws is easy to adjust as both use screw mechanism. As the pitch of lag screw of DHS is 3.5 mm, half turn which is the minimum unit of adjustment the operator can make is 1.75 mm. Although the cannulated screws are generally manufactured in the unit of 5 mm, surgeon can control the depth of insertion in analogue scale and has more option with washers to adjust the depth. However, the depth of bolt in FNS is difficult to finely control as the length of the bolt is in units of 5 mm. The manufacturer recommended the subtraction of 5 mm from the measured depth read on the direct measuring device and choose the next shorter bolt size [12]. For example, if the measured depth was 102 mm in measuring device after insertion of tip of central guide wire into the subchondral bone, it is recommended to choose 95 mm bolt. According to this manufacturer`s guideline, the implant is positioned within 20 mm of the tip-apex distance (TAD) after surgery [13]. However, TAD is a criterion applied in fixation of DHS for intertrochanteric fractures to lower failure rates, and it has not been demonstrated whether it can be applied to femoral neck fracture using FNS. Also, it is a concern whether the method will ensure the insertion of bolt into sufficient depth and stable fixation of femoral neck fractures.

Thus, this study introduces a method to control the depth of FNS bolt in millimeter unit in patients with femoral neck fracture.

## Technical note

The technique to be introduced in this study can be applied to all fixations of femoral neck fracture using FNS, and all the indications and contraindications of this technique follows those indicated on the surgical manual.

### Operative technique

**Fig. 2:**
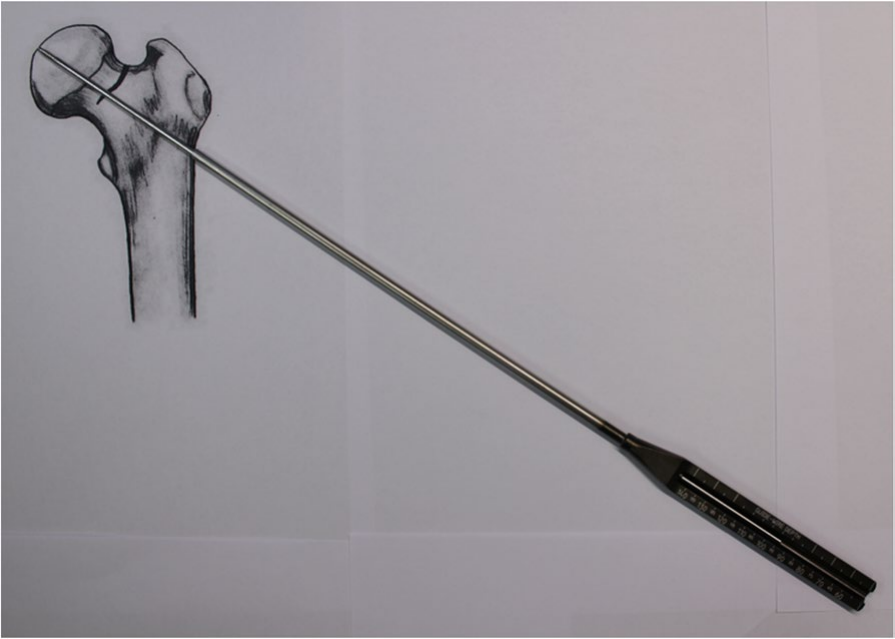
After the depth of central guide wire embedded in the femoral bone is measured, the size of bold is selected which is next longer construction size. The photographs were taken by the authors

After reduction of the femoral neck fracture, the central guide wire is placed into the surgeon’s targeted position under the guidance of image intensifier (Fig. 2) (Table 1). After the depth of guide wire embedded in the subchondral bone is measured, the next longer construct size is selected. For example, should the depth guide indicate a measurement between 97 and 98 mm, then a 100 mm-length bolt would be considered (in comparison to the manufacturer’s guideline’s recommendation of a 90 mm-length bolt).

**Table 1:** Comparison between the surgical procedure presented by the manufacturer and the surgical procedure presented by the author

Manufacturer`s guideline	Author`s method
1. Central guide wire insertion	1. Central guide wire insertion
2. depth measuring: 98mm	2. depth measuring: 98mm
3. size of bolt: 90mm	3. size of bolt: 100mm (one size longer than the measured depth)
4. reaming of 90mm reamer	4. 2 step reaming first 90mm reamer (same with manufacturer`s guideline) second 100mm reamer to target depth under image intensifier (same with selected bolt size)
5. plate, bolt, insertion handle assembled by black screw	5. plate, bolt, insertion handle assembled by black screw
6. insertion of bolt	6. insertion of bolt
	7. some untightening of black screw and make contact between plate and femoral cortex
7. insertion of distal locking screw	8. insertion of distal locking screw
	9. some retraction of bolt treated by retightening of black screw and tapping by impactor
8. insertion of antirotation screw	10. insertion of antirotation screw
9. fracture site compression	11. fracture site compression
10. removal of insertion handle	12. removal of insertion handle

The operator can control the depth of reaming by dividing the reaming procedure into two steps (as to the manufacturer`s guideline recommendation: single 90 mm reaming). Continuing the former example, the reamer of the first reaming step should be set at 90 mm. The first reaming is to countersink the lateral cortex of femur. By sliding the reamer-component over the drill bit into the remarked numbers, the limit of reamer tip advancement can be controlled by the remarked numbers. Assembling the reamer to set the depth of reaming for 10 mm less than the implant size, the surgeon can achieve countersinking for the barrel of the plate without penetration of articular surface.

To make room for a deeper insertion of the 100 mm-length bolt to the targeted position after the first reaming using a 90 mm-length bolt, the final advancement of reamer tip is adjusted finely on the second reaming step. After the reamer was re-assembled to indicating number on the reamer as the same number of the implant size (Continuing the former example, the reamer of the second reaming step should be set at 100 mm.), the reamer without power tool is pushed manually back into the pre-reamed hole to touch the blind end of reamed hole with reamer tip. The manual advancement and rotation in counterclockwise of the reamer without power tool ease the reinsertion of reamer without unintended harm of osteoporotic trabeculae. After reaching the end of blind pipe, the reamer is assembled to the power tool and image intensifier is used to estimate the remaining distance to reach the target. Under the guidance of image intensification, the reamer is advanced to the target carefully.

**Fig. 3:**
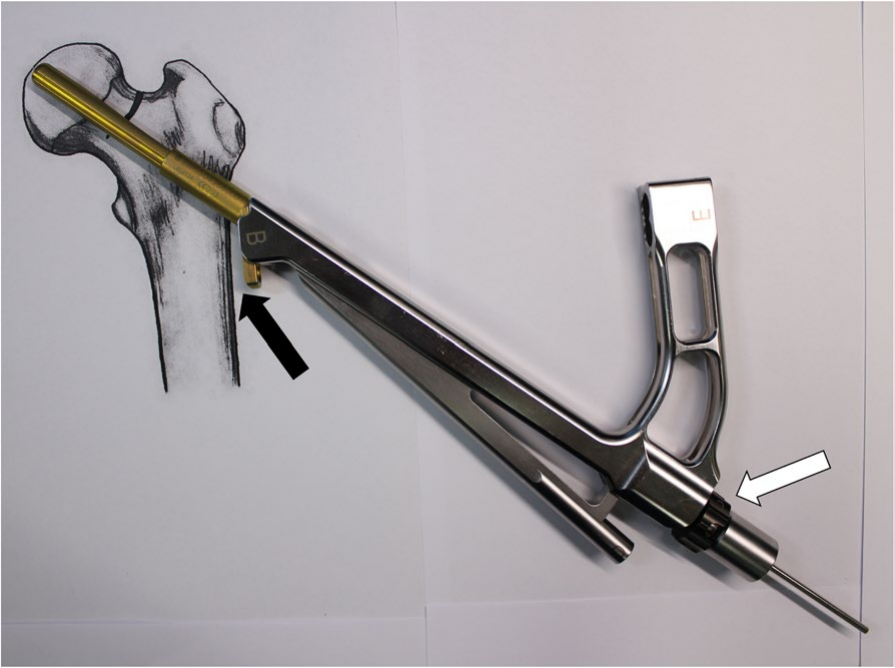
After reaming to target depth under a fluoroscopy, insert bolt and plate by insertion handle. The white arrow indicates black screw which combines the insertion handle, plate, and bolt. The black arrow indicates that plate does not contact with lateral cortex of femur. The photographs were taken by the authors

**Fig. 4:**
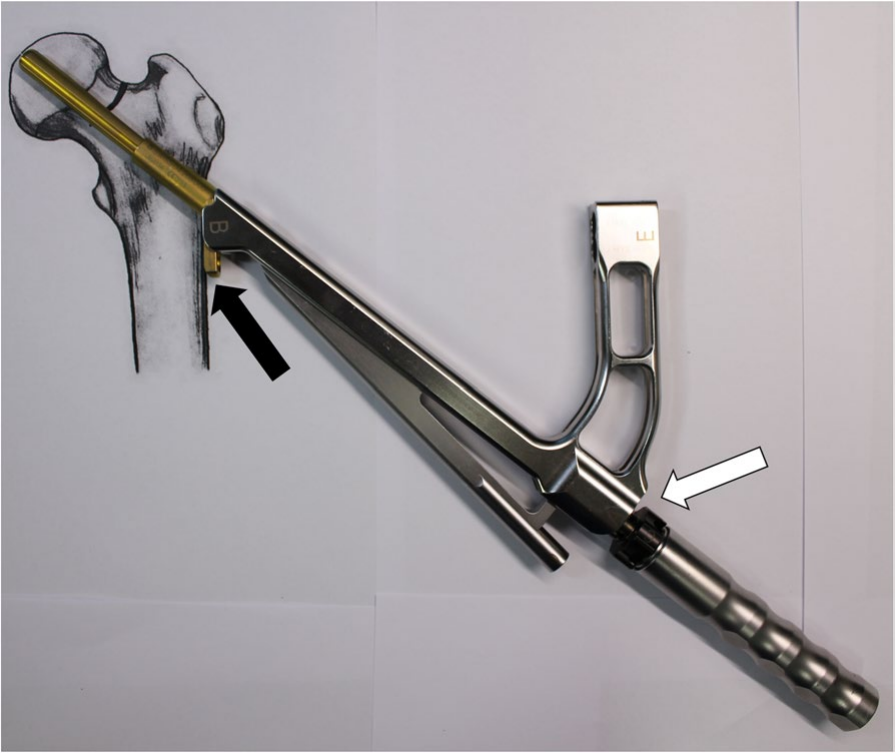
Loose the black screw and impact the plate using cylinder impactor. It makes contact between plate and lateral femoral cortex. The white arrow indicates loosen black screw and the black arrow indicates contact between plate and lateral femoral cortex. The photographs were taken by the authors

**Fig. 5:**
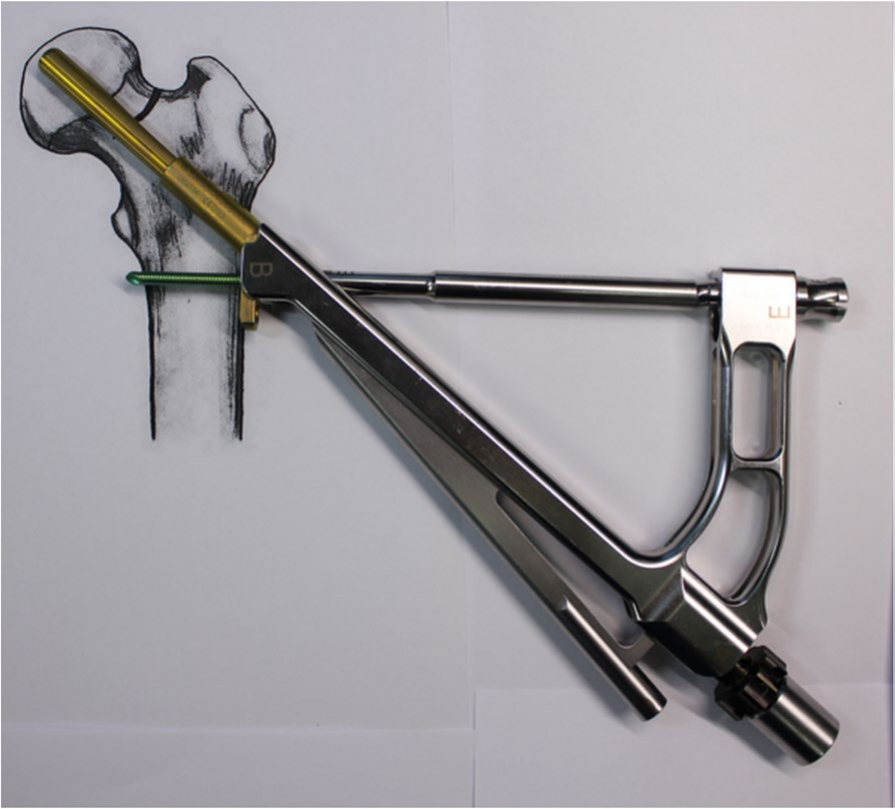
Insert distal locking screw, taking care not to insert eccentrically into the subtrochanteric area. The photographs were taken by the authors

**Fig. 6:**
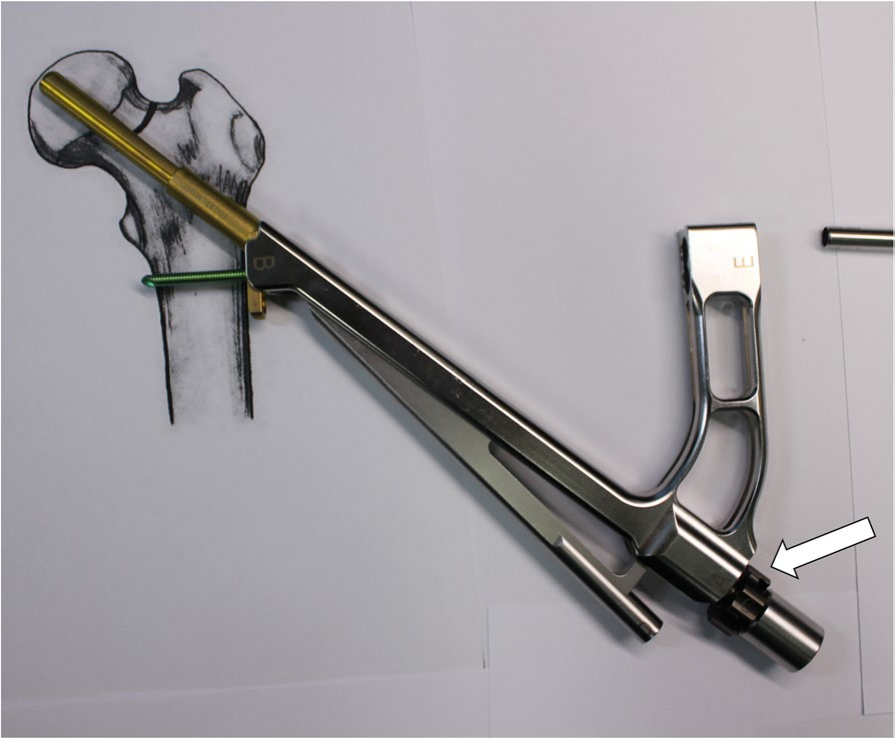
During the impact for contact between plate and lateral femoral cortex, bolt could be pulled out from target depth. In this case, bolt can be inserted back to the target position by tightening the black screw. The white arrow indicates the tightened the black screw. The photographs were taken by the authors

**Fig. 7:**
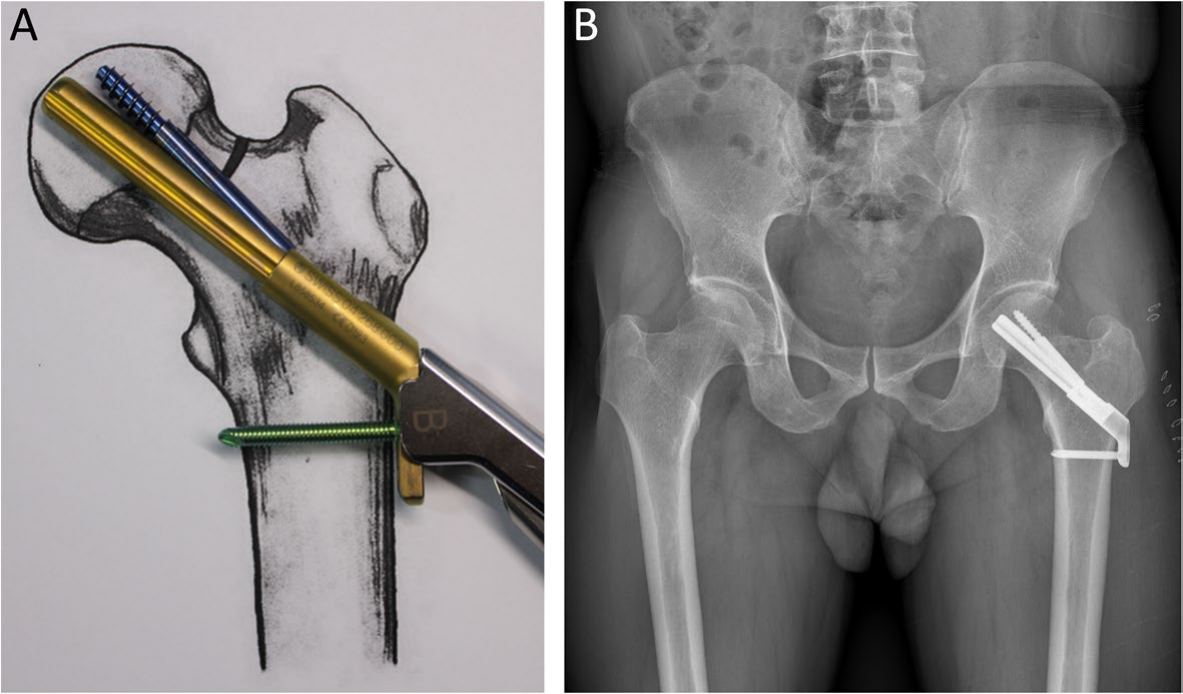
**A** Insert antirotation screw. **B** An immediate postoperative radiograph of in a 54-year-old male patient with femoral neck fracture who was treated with the Femoral neck system. The photographs were taken by the authors

**Fig. 8:**
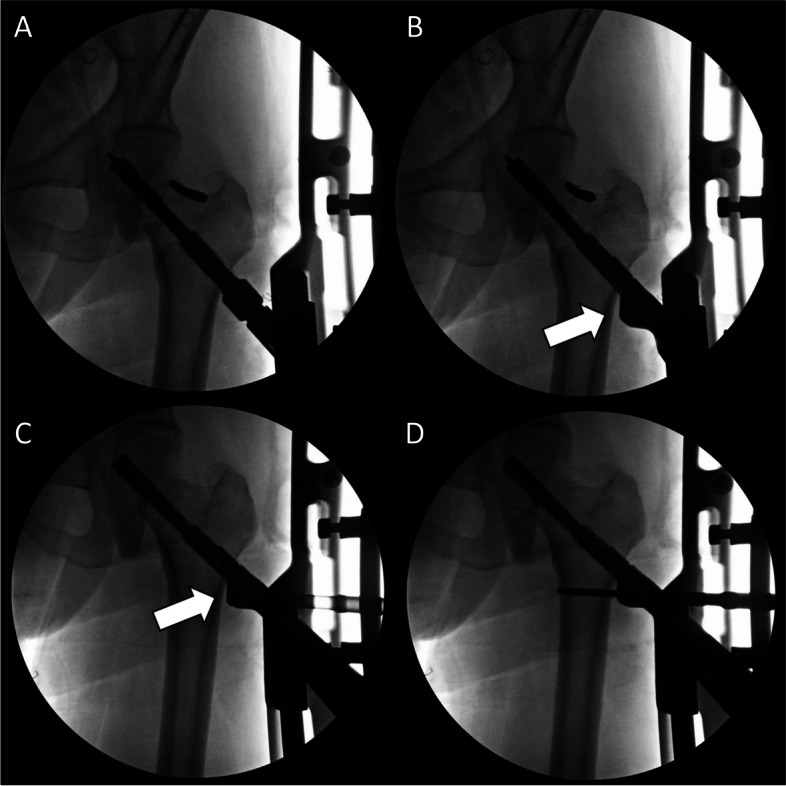
Indraoperative radiographs of the case shown in Fig. 7B show the surgical procedure for femoral neck fracture using the Femoral neck system. **A** After closed reduction using a Steinmann pin, a guide pin was inserted and reaming was performed. **B** The femoral neck system inserted with an insertion handle. According to our technique, the plate could not be contacted with the lateral femoral cortex (white arrow). **C** To reduce gap between plate and lateral femoral cortex (white arrow), the black screw was loosened and the plate was impacted. **D** After then, locking screw was inserted. The photographs were taken by the authors

The plate, bolt, insertion handle, and insert are assembled by tightening of black screw as the surgical manual. After then, FNS is inserted. The cylinder impactor can be used to tap manually the plate on to the bone. When the tip of bolt reaches the target depth, plate does not touch the lateral surface of femoral cortex as the length of bolt is longer than the depth of reaming (Fig. 3). By untightening of the black screw, bolt is retracted backward (Fig. 4). After retraction of bolt, plate could contact the lateral surface of femoral cortex with tapping. After removal of central guide wire, distal locking screw is inserted, taking care not to insert eccentrically into the subtrochanteric area (Fig. 5). During this procedure, the bolt could be retracted more than intended. If black screw is re-tightened, the bolt protracts to reach the base of reamed bone (Fig. 6). After the surgeon confirms that the tip of bolt is positioned on the target and the plate is set on the lateral cortex with the image intensifier, the anti-rotation screw is inserted (Fig. 7). Inter-fragmentary compression is up to surgeon’s decision. Intraoperative radiographs of the case shown in Fig. 7B demonstrate clinical application of our technique (Fig. 8).

From September 2019 to September 2020, investigation for radiographs was conducted on 14 patients who were hospitalized for femoral neck fracture at our hospital and underwent surgery using FNS. All radiographs were retrieved from a picture archiving communication system of M-view (version 5.4.10.38, Infinitt Healthcare Co., Seoul, South Korea). All radiographs were calibrated before their evaluation using the known diameter of the bolt (10 mm). In both hip AP with 15˚ internal rotation view and translateral view of affected hip, TAD was measured for the tip of bolt [13].

## Results

The average TAD measured in the cases that performed surgery with the manufacturer's surgical technique (10 patients) was 16.3 mm (range 14.6 to 18.1 mm), and the average TAD measured in the cases who performed surgery with the technique suggested in this study (4 patients) was 7.2 mm (range 6.1 to 12.2 mm). On the Mann–Whitney U test, TAD of the group that underwent surgery with the technique suggested in this study was significantly shorter than that of the group with the method of manufacturer’s guide (*p* < 0.05).

## Discussion

Previous comparative biomechanical studies between FNS and other implants in cadaveric femoral neck fracture showed that the FNS can provide superior stability to the femur neck fractures [9, 14]. Stoffel et al. performed a biomechanical test after insertion of DHS, MCS and FNS in a cadaveric femoral neck fracture model with posteromedial bone defect [9]. The results of their study showed that FNS provided superior stability compared to MCS and had comparable stability to that of DHS. Schopper et al. performed a biomechanical test by inserting FNS and Hansson pins in the cadaveric model of Pauwels type II [14]. They reported that FNS was more resistant in varus deformation and less sensitive to variations in implant placement compared to Hansson pins. And, they insisted that FNS with diverging lag screw has superiority in femoral neck fracture in terms of less neck shortening. In both studies, the length of the bolt during FNS fixation was determined to make the TAD be within 20 mm which is originally from the evaluation method of DHS or proximal femoral nails in intertrochanteric fractures [13]. Surgery according to the surgical guide of manufacturer also makes the TAD within 20 mm. However, although FNS is morphologically similar to DHS, it does not seem to be proven whether it is reasonable to apply the same evaluation method for the position of FNS in femoral neck fractures which has a shorter length of proximal fragment than that of intertrochanteric fractures.

With MCS, the length as well as the diameter and position of the screw are important [15, 16]. Lindequist reported that the screws must be located within 3 mm of the femoral head cortex to achieve cortical support [16]. They recommended the insertion of screws with enough length to increase mechanical stability. Rau et al. showed that the insertion of the DHS screw into the subchondral region of the femoral head provided satisfactory clinical results of the femoral neck fractures [17]. They also reported that the depth of the DHS screw is an important factor related to the unsatisfactory results. Therefore, subchondral implant anchorage in femoral head by a longer implant plays an important role in stabilizing the femoral neck fractures. However, it is difficult to achieve an ideal depth of FNS bolt following the manufacturer's surgical guide alone. We believe the technique of this study can help to overcome the limitation of the implant manufactured in 5 mm unit and additionally contribute to increase fixation stability.

**Fig. 9:**
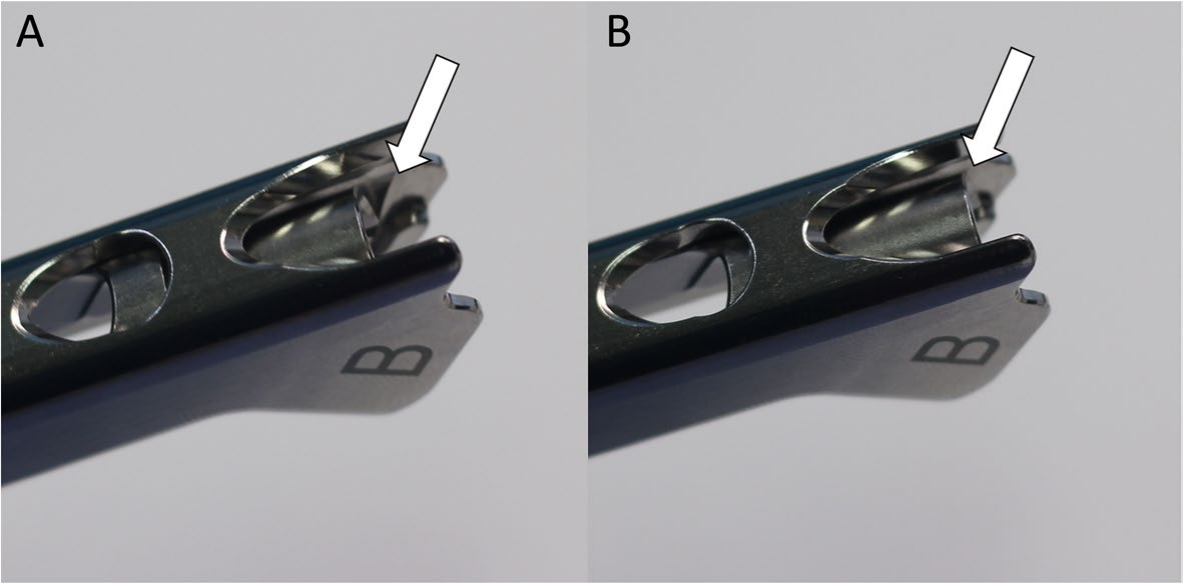
The radiographs show the connection between plate of FNS and insertion handle by tightening of black screw. **A** The white arrow indicates the slit for connecting with plate. If the black screw is completely loosened with insertion hand, plate can be disconnected from insertion handle. **B** The white arrow means that plate slit is blocked by metal portion of black screw. If the black screw is tightened even a little, plate cannot be disconnected from handle. The photographs were taken by the authors

**Fig. 10:**
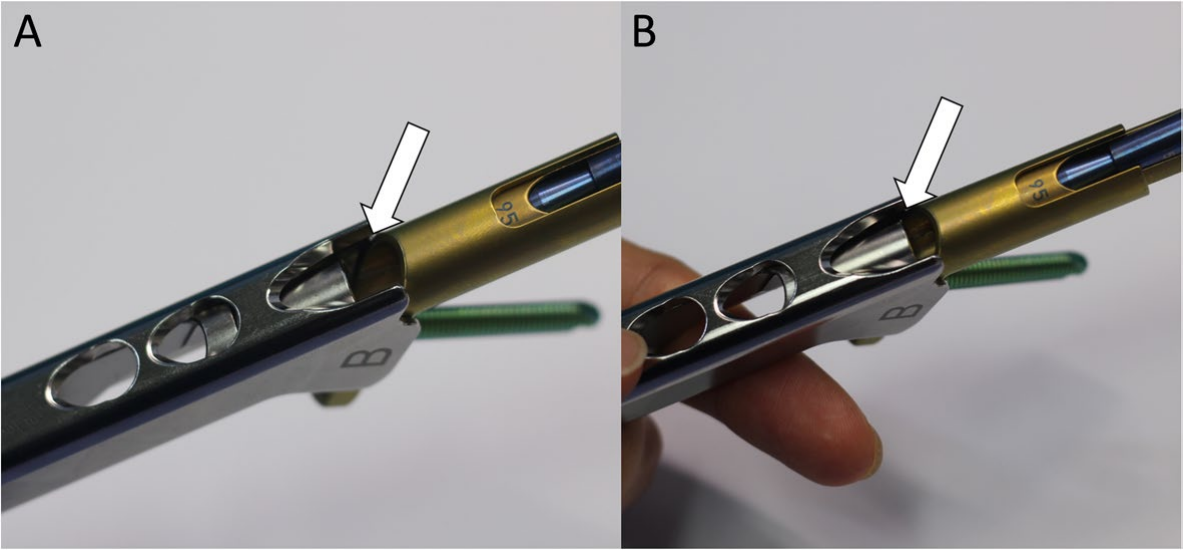
**A** The white arrow show plate slit is not blocked by metal when the black screw is completely loosened. **B** The white arrow show plate slit is blocked by metal when the black screw is tightened even a little. It makes prevention of disconnection between plate and handle. The photographs were taken by the authors

One concern of the technique is the disengagement between insertion handle and FNS while inserting the assembly of implant and instruments. Theoretically, the disengagement before the insertion of distal locking screw and antirotation screw can block right insertion of screws. However, as complete disengagement requires complete clearance of the insert from the plate and the insertion handle holds the plate tight, the implant cannot disengage from the insertion handle while using our technique. Also, the thread of black screw is on the track of the thread on the insertion handle even in a loose manner, the connecting part to the bolt of the insert block the plate slit form the disengagement (Figs. 9 and 10).

The limitation of this study is that it has not been proven that a difference in fixation of several millimeters causes a clinical difference in treatment of femoral neck fracture using FNS, and the number of cases is small. Also, there was a lack of consideration for other factors that could influence clinical results, such as femoral neck shortening. Further research on this is needed to delineate the clinical difference.

## Conclusion

In conclusion, even though the bolt of FNS is manufactured in the unit of 5 mm, the technique proposed in this study helps surgeons to adjust the depth of bolt for the fixation of femoral neck fracture using FNS.

## Abbreviations

DHS: Dynamic hip screw
FNS: Femoral Neck System
MCS: Multiple cannulated screws
TAD: Tip-apex distance
